# Reverse Evidence-Based Dentistry: An Innovative Approach to Dental Education and Clinical Practice

**DOI:** 10.7759/cureus.94156

**Published:** 2025-10-08

**Authors:** Andrea Mascolo, Oana Dinculescu, Maria Mezetti, Gilbert Mercieca, Francesca Busuttil

**Affiliations:** 1 EIMS Research Hub, European Institute for Medical Studies (EIMS), St. Julian’s, MLT; 2 EIMS Research Hub, European Institute for Medical Studies (EIMS), St. Julian's, MLT

**Keywords:** critical appraisal, dental education, evidence-based dentistry (ebd), interdisciplinary curriculum, reverse evidence-based dentistry

## Abstract

Evidence-Based Dentistry (EBD) has become a cornerstone of modern dental practice, integrating current research evidence with clinical expertise. Despite its recognized value, barriers such as limited time, inadequate training, and reliance on non-scientific or market-driven sources continue to hinder its consistent application. To address these challenges, we introduce the concept of Reverse Evidence-Based Dentistry (Reverse EBD), defined as a structured analytical process that begins with real-world clinical practices or historical observations and works backward to identify the scientific evidence supporting them. In contrast to traditional EBD, which applies published evidence to guide clinical decision-making, Reverse EBD retraces the evidence pathway from practice to research, promoting reflection and analytical rigor. This complementary approach encourages critical thinking, deepens understanding of how clinical protocols evolve, and fosters the ability to differentiate between evidence-based and commercially driven recommendations.

Drawing on examples from periodontal laser therapy and platelet-rich fibrin, Reverse EBD has demonstrated educational value by stimulating critical appraisal among students and clinicians. The concept has been integrated into the Bachelor of Science (Honours) in Dental Science at the European Institute of Medical Studies (Malta), within a neutral pedagogical framework designed to link theory, clinical reasoning, and interdisciplinary learning. Early implementations have shown increased student engagement and critical reasoning skills compared to traditional EBD instruction. Reverse EBD thus represents a promising educational and analytical model for reinforcing evidence-based practice and preparing future dental professionals for innovation, research, and critical inquiry.

## Editorial

Evidence-Based Dentistry (EBD) involves the integration and interpretation of the most current research evidence with personal experience. First cited in professional dental literature in 1995 [1], EBD allows dentists, as well as academic researchers, to keep abreast of new developments and make decisions that improve clinical practice. Today, most dentists are familiar with EBD [2] and generally support its implementation [2].

However, despite its benefits, barriers to EBD implementation remain, including insufficient time, inadequate training, and a perceived lack of relevance of the evidence to everyday practice [3]. Dentists often rely on colleagues, key opinion leaders, company sales representatives or social media forums to gain new information [4]. However, these sources are not always up-to-date or correct, and may provide financially motivated advice. Social media forums lack quality control, and yet due to the visibility of cases posted, they are likely to have an overinflated effect on practice [4]. Busy clinicians need access to selective, efficient and patient-driven research. It is crucial for clinicians to be able to assess the level of evidence of publications to formulate an evidence-based treatment plan and provide patients with accurate, current, and trustworthy information.

In light of these challenges, this commentary introduces the concept of Reverse Evidence-Based Dentistry (Reverse EBD), defined as a structured analytical process that inverts the conventional EBD sequence. Unlike traditional EBD, which begins with published evidence to inform clinical decisions, Reverse EBD starts from real-world clinical practices or historical observations and systematically traces backward to identify and evaluate the supporting scientific evidence. This approach not only clarifies the evidence base behind established protocols but also trains learners to discern gaps between practice and research, fostering reflective, critical, and evidence-aware clinical reasoning.

EBD has a significant influence on oral healthcare, yet persistent barriers - such as limited time, perceived complexity, and insufficient translation of evidence into practice - continue to hinder its consistent adoption. Reverse EBD is proposed as a complementary framework designed to address these limitations. Unlike conventional EBD, which follows a top-down model that begins with published evidence and applies it to clinical settings, Reverse EBD starts from real-world clinical practices and works backward to identify and evaluate the supporting scientific rationale. This inversion of the evidence pathway promotes active engagement, contextual understanding, and reflective inquiry, helping clinicians and students critically assess both established and emerging treatment protocols. By fostering a dynamic link between observation and evidence, Reverse EBD bridges the gap between theory and practice, making evidence-based reasoning more accessible and applicable to everyday clinical decision-making.

At the European Institute of Medical Studies (EIMS), Reverse EBD has been integrated into undergraduate teaching within a neutral, non-promotional pedagogical framework aimed at developing analytical and reflective competencies. This educational implementation demonstrates how Reverse EBD can strengthen evidence-based thinking while preserving academic independence and methodological rigor.

Additionally, EIMS has introduced the concept of Reverse EBD through an engaging and easily reproducible process that builds on foundational clinical skills. By readapting the concept of reverse engineering to dentistry, Reverse EBD is defined as a structured analytical process through which one seeks to understand, by means of deductive reasoning, how an established clinical or educational procedure has been developed and supported by scientific evidence. This perspective enables learners to reconstruct the rationale behind existing practices and critically evaluate their evidence base.

Undergraduate students at EIMS received foundation training in EBD principles and then applied Reverse EBD to a foundational unit of their curriculum: History of Medicine. As part of this exercise, students virtually examined Mayan human skulls exhibited at the Peabody Museum at Harvard University, questioning how three shell fragments were integrated into the mandible. Additionally, they also considered archaeological evidence from other cultures. They explored how the Etruscans in Italy and the Ancient Egyptians used cast iron, copper and even oxen bone to create the first tooth substitutes. This exercise allowed students to link different units and develop a critical assessment of their learning. By examining historical practices through the lens of modern evidence-based principles, students gained insights into the evolution of dental practices and the importance of EBD in modern dentistry.

The Reverse EBD approach can also be applied to clinical protocols beyond historical contexts. For instance, while laser protocols have been strongly promoted for the treatment of periodontal disease and peri-implantitis [4], students applying the Reverse EBD process discovered differing recommendations. In fact, the S3 Level Clinical Practice Guideline for the treatment of Stage I-III periodontitis [5], published by the American and European Academies of Periodontology, do not recommend use of lasers. This critical reverse process stimulates learners to adopt a more conscious and evidence-based approach to clinical practice, encouraging them to evaluate and validate current protocols against the best available evidence.

Another example of the Reverse EBD process applied to clinical protocols is for the use of platelet-rich fibrin (PRF). PRF, used alone or in combination with grafts or other biomaterials, has shown regenerative effectiveness in different clinical fields [6]. The Reverse EBD exercise allowed students to assess higher levels of evidence related to specific procedures. For instance, a meta-analysis on the effectiveness of PRF as an adjunctive material to bone graft in maxillary sinus augmentation demonstrated no clear advantage or disadvantage in using PRF preparations or their by-products alongside bone graft materials [7]. This exercise helps students critically evaluate evidence, understand the complexities of clinical protocols, and identify alternative protocols equally supported by evidence.

In forensic dentistry, it has been noted that EBD is sometimes employed to justify procedures rather than to genuinely guide best practices. This misuse of EBD is a concern that is acknowledged within both forensic and general dental literature [8]. In this context, as well as in clinical dentistry, the Reverse EBD process could be a valuable tool for critically evaluating evidence and proposed protocols. By encouraging a more thorough examination of clinical practices, the Reverse EBD approach may result in an improved understanding and application of evidence-based practices in clinical settings. Furthermore, through Reverse EBD, clinicians may identify procedures that may be more compatible with variables such as their level of clinical experience.

Over the last 30 years, the contents, methods and assessment of EBD education have been widely studied and discussed. A recent scoping review revealed that the current literature focuses mainly on teaching of critical appraisal skills, traditional teaching methods, and short-term outcome assessments [9]. To address these gaps, future research should explore comprehensive educational models, multifaceted teaching approaches, and validated outcome assessment tools [9]. Among the strategies described for effectively teaching EBD in undergraduate courses, the following have been highlighted: designing continuing and frequent dental education courses, establishing collaborative student research groups, utilising online tools for EBD education, and dividing EBD courses into shorter modules [10]. Teaching strategies that incorporate clinical experiences tend to be more effective in fostering critical thinking and application of EBD principles. Implementing EBD in the foundational years of dental education, even when students have limited prior dental knowledge, can encourage early development of analytical and reflective skills essential for evidence-based practice.

Furthermore, the concept of Reverse EBD has been formally incorporated into the newly accredited Bachelor of Science (Honours) in Dental Science (180 ECTS, Malta Qualifications Framework (MQF) Level 6) at EIMS, Malta. The programme adopts an interdisciplinary curriculum combining dental science with biomedical engineering, health IT, and research methodology, offering a neutral pedagogical framework that integrates Reverse EBD as both a theoretical and practical approach to developing analytical and reflective competencies.

This programme combines dental science with biomedical engineering, health IT, and advanced research methodologies, preparing graduates for careers at the intersection of clinical practice, research, and healthcare innovation [11,12]. Within this curriculum, Reverse EBD is applied not only as a theoretical framework but also as a practical pedagogical tool, enabling students to critically analyze both historical practices and contemporary protocols. This integration illustrates how Reverse EBD supports the development of innovative educational approaches and the enhancement of critical appraisal competencies among future dental professionals.

The introduction of Reverse EBD encourages clinicians to critically assess the level of evidence in publications and to differentiate between evidence-based guidelines and market-driven protocols. By focusing on protocols supported by robust evidence, and using a hierarchical pyramid for initial evaluation, clinicians can ensure that they are applying the most appropriate and effective treatments based on solid research and critical assessment of their own knowledge and skills. The Reverse EBD framework enhances the understanding and practical application of evidence-based practices by promoting a systematic, reflective approach to evaluating existing protocols and identifying the scientific rationale behind them.

Reverse EBD represents a complementary approach to traditional EBD, fostering critical appraisal of both historical and contemporary protocols. Its integration into the innovative BSc (Hons) Dental Science programme demonstrates its potential as both a pedagogical strategy and a clinical mindset, supporting the development of critically minded, research-driven dental professionals.
